# Supplementary material for: Effect of antithrombotic stewardship on the efficacy and safety of antithrombotic therapy during and after hospitalization
Source: PLoS One. 2020 Jun 25;15(6):e0235048. doi: 10.1371/journal.pone.0235048 (PMC7316339; doi:10.1371/journal.pone.0235048)
Supplement: S1 Table — e-GFR estimated glomerular filtration rate, INR International Normalized Ratio. (PDF) [file pone.0235048.s002.pdf]

Table S1 Data collection

**Part**	**Data content**
Patient data	Patient ID
	Date of birth
	Gender
	Bodyweight on the day of hospitalization
	Date of hospitalization
	Date of hospital discharge
	Type of hospital (University Medical Center/general hospital)
	Bleeding in history (yes/no)
	Thrombotic event in history (yes/no)
	Readmission within 3 months after discharge (yes/no)
Interventions	Surgical procedure (yes/no)
Clinical outcomes	Bleeding (major and non-major) event(s) during hospitalization
	Bleeding (major and non-major) event(s) within 3 months after hospitalization
	Severity of bleeding event(s)
	Location of bleeding event(s)
	Thrombotic event(s) during hospitalization
	Thrombotic event(s) within 3 months after hospitalization
	Severity of thrombotic event(s)
	Location of thrombotic event(s)
	Length of hospitalization
	All-cause mortality during and within 3 months after hospitalization
Costs	Time spent on labour S-team in-hospital costs (S-team meetings, medication reviews by (hospital) pharmacists, patient counselling, drafting and maintenance of anticoagulant therapy protocols and education)
	Costs for hospitalization days
	Costs for non-major and major bleeding events during and after hospitalization
	Costs for thrombotic events during and after hospitalization
Medication data	Type of anticoagulant therapy
	➢ Vitamin K antagonist
	➢ Direct oral anticoagulant
	➢ Low-molecular-weight-heparin
Clinical chemistry data	Laboratory values
	➢ e-GFR (ml/min/1.73m^2) on the day of hospitalization
	➢ INR
	➢ Haemoglobin (mmol/L)

e-GFR estimated glomerular filtration rate, *INR* International Normalized Ratio
