# Supplementary material for: Effect of antithrombotic stewardship on the efficacy and safety of antithrombotic therapy during and after hospitalization
Source: PLoS One. 2020 Jun 25;15(6):e0235048. doi: 10.1371/journal.pone.0235048 (PMC7316339; doi:10.1371/journal.pone.0235048)
Supplement: S2 Table — These bleeding and thrombotic events occurred in 135 patients in the usual care period and 124 patients in the intervention period. (PDF) [file pone.0235048.s003.pdf]

Table S2 Characterization of all bleeding and thrombotic events before and after implementation of the multidisciplinary antithrombotic team

Bleeding and thrombotic events	Usual care period (*n*=941) N (%)	Intervention period (*n*=945) N (%)
**Bleeding events***	**136 (14.5)**	**130 (13.8)**
Severity of bleeding		
Major bleeding	89 (9.5)	68 (7.2)
Non-major bleeding	46 (4.9)	62 (6.6)
Unknown	1 (0.1)	-
Location of bleeding		
Surgical site bleeding	63 (6.7)	51 (5.4)
Gastrointestinal bleeding	25 (2.7)	25 (2.6)
Urogenital bleeding	16 (1.7)	18 (1.9)
Oral and nasal bleeding	11 (1.2)	12 (1.3)
Other	21 (2.2)	24 (2.5)
**Thrombotic events***	**25 (2.7)**	**20 (2.1)**
Severity of thrombotic event		
Fatal	1 (0.1)	0 (0.0)
Non-fatal	24 (2.6)	20 (2.1)
Location of thrombotic event		
Ischemic stroke	9 (1.0)	6 (0.6)
Pulmonary embolism	5 (0.5)	6 (0.6)
Leg thrombosis	4 (0.4)	0 (0.0)
Other	7 (0.7)	8 (0.8)

*These bleeding and thrombotic events occurred in 135 patients in the usual care period and 124 patients in the intervention period.
