# Supplementary material for: Effect of antithrombotic stewardship on the efficacy and safety of antithrombotic therapy during and after hospitalization
Source: PLoS One. 2020 Jun 25;15(6):e0235048. doi: 10.1371/journal.pone.0235048 (PMC7316339; doi:10.1371/journal.pone.0235048)
Supplement: S3 Table — OR odds ratio, 95% CI 95% confidence interval. (PDF) [file pone.0235048.s004.pdf]

Table S3 Proportion of patients with a composite end point consisting of ≥1 bleeding or ≥1 thrombotic event during and 3 months after hospitalization

	**Usual care period** (*n*=941)	**Intervention period** (*n*=945)	**OR (95% CI)**	**ORadj (95% CI)**
Bleeding and thrombotic events during hospitalization	73/941 (7.8%)	65/945 (6.9%)	0.88 (0.62-1.24)	0.90 (0.63-1.30)
Bleeding and thrombotic events 3 months after hospitalization	66/941 (7.0%)	66/945 (7.0%)	1.00 (0.70-1.42)	0.95 (0.66-1.37)

OR odds ratio, *95% CI* 95% confidence interval; ORadj, adjusted for characteristics differing between usual care and intervention period (bleeding in history and treatment with VKAs, DOACs or LMWHs)
