# Supplementary material for: Effect of antithrombotic stewardship on the efficacy and safety of antithrombotic therapy during and after hospitalization
Source: PLoS One. 2020 Jun 25;15(6):e0235048. doi: 10.1371/journal.pone.0235048 (PMC7316339; doi:10.1371/journal.pone.0235048)
Supplement: S4 Table — (PDF) [file pone.0235048.s005.pdf]

Table S4 Causes of death

**Cause of death**	**Usual care period (*n*=108)**<br>**N (%)**	**Intervention period (*n*=81)**<br>**N (%)**
Cancer	38 (35.2)	27 (33.3)
Cardiovascular disease	24 (22.3)	22 (27.2)
Cerebrovascular accident	1 (0.9)	0 (0)
Diabetes, blood and endocrine disease	4 (3.7)	3 (3.7)
Infectious diseases	10 (9.3)	12 (14.8)
Kidney disease	9 (8.3)	1 (1.2)
Liver disease	1 (0.9)	1 (1.2)
Multiple organ failure	4 (3.7)	9 (11.2)
Other	5 (4.6)	0 (0)
Respiratory diseases	9 (8.3)	6 (7.4)
Unknown	3 (2.8)	0 (0)
