# Supplementary material for: Effect of antithrombotic stewardship on the efficacy and safety of antithrombotic therapy during and after hospitalization
Source: PLoS One. 2020 Jun 25;15(6):e0235048. doi: 10.1371/journal.pone.0235048 (PMC7316339; doi:10.1371/journal.pone.0235048)
Supplement: S5 Table — (PDF) [file pone.0235048.s006.pdf]

Table S5 Sensitivity analysis for costs of non-major bleeding

	Usual care period	Intervention period
	Mean costs per patient (€)	Mean costs per patient (€)
**Erasmus Medical Center**		
Labour costs S-team	-	€ 44.80
S-team meetings	-	€ 0.30
Medication reviews	-	€ 32
Patient empowerment	-	€ 12
Maintenance of anticoagulant therapy protocols and education	-	€ 0.50
Costs for hospitalization days	€ 9360	€ 8580
Costs for bleeding	€ 899	€ 869
Non-major bleeding with hospitalization	€ 194	€ 268
Non-major bleeding without hospitalization	€ 28	€ 25
Major bleeding	€ 677	€ 576
Costs for thrombotic events	€ 169	€ 143
Arterial thrombosis	€ 81	€ 71
Deep vein thrombosis	€ 56	€ 56
Pulmonary embolism	€ 32	€ 16
**Total costs (p= 0.27)**	**€ 10430**	**€ 9640**
**Reinier de Graaf Gasthuis**		
Labour costs S-team	-	€ 35.50
S-team meetings	-	€ 0.50
Medication reviews	-	€ 23
Patient empowerment	-	€ 10
Maintenance of anticoagulant therapy protocols and education	-	€ 2
Costs for hospitalization days	€ 3970	€ 3570
Costs for bleeding	€ 584	€ 483
Non-major bleeding with hospitalization	€ 121	€ 166
Non-major bleeding without hospitalization	€ 21	€ 42
Major bleeding	€ 442	€ 275
Costs for thrombotic events	€ 109	€ 77
Arterial thrombosis	€ 41	€ 51
Deep vein thrombosis	€ 19	€ 10
Pulmonary embolism	€ 49	€ 16
**Total costs (p= 0.07)**	**€ 4660**	**€ 4170**
