# Supplementary material for: Effect of antithrombotic stewardship on the efficacy and safety of antithrombotic therapy during and after hospitalization
Source: PLoS One. 2020 Jun 25;15(6):e0235048. doi: 10.1371/journal.pone.0235048 (PMC7316339; doi:10.1371/journal.pone.0235048)
Supplement: S1 File — (PDF) [file pone.0235048.s007.pdf]

RESEARCH PROTOCOL

Antithrombotic stewardship: a multidisciplinary team approach towards improving antithrombotic therapy during and after hospitalization

**Protocol ID**	**NL53725.078.15**
**Short title**	**Antithrombotic stewardship**
**EudraCT number**	**-**
**Version**	**1.0**
**Date**	**29-05-2015**
**Coordinating investigator/project leader**	**Dr. P.M.L.A. van den Bemt**<br>Hospital pharmacist-clinical pharmacologist/epidemiologist<br>*T:* 010-7033202 | *F:* 010-7032400| *E:* p.vandenbemt@erasmusmc.nl<br>Erasmus MC<br>Apotheek<br>Thema Diagnostiek & Advies<br>Postbus 2040<br>3000 CA ROTTERDAM<br><br>**Dr. M.J.H.A. Kruip**<br>Internist-hematologist<br>*T:* 010-7033123 | *E:* m.kruip@erasmusmc.nl<br>Erasmus MC<br>Hematologie<br>Postbus 2040<br>3000 CA ROTTERDAM<br><br>**Dr. J. Diepstraten**<br>Hospital pharmacist<br>T: 015-2605079 | *E:* j.diepstraten@rdgg.nl<br>Reinier de Graaf ziekenhuis<br>Ziekenhuisfarmacie<br>Postbus 5011<br>2625 AD Delft<br><br>**Dr. R. Brouwer**<br>Internist-hematologist<br>T: 015-2603296 | *E:* rolf.brouwer@rdgg.nl<br>Reinier de Graaf ziekenhuis<br>Hematologie<br>Postbus 5011<br>2625 AD Delft

	**Prof. dr. F.W.G. Leebeek**<br>Internist-hematologist<br>*T:* 010-7031672 | *E:* f.leebeek@erasmusmc.nl<br>Erasmus MC<br>Hematologie<br>Postbus 2040<br>3000 CA ROTTERDAM<br><br>**Prof. dr. A.G. Vulto**<br>Hospital pharmacist-clinical pharmacologist<br>*T*: 010 703 32 02 | *E*: a.vulto@erasmusmc.nl<br>Apotheek<br>Thema Diagnostiek & Advies<br>Postbus 2040<br>3000 CA ROTTERDAM
**Principal investigator(s) (in Dutch: hoofdonderzoeker/ uitvoerder)**	**Dr. P.M.L.A. van den Bemt,** hospital pharmacist-clinical pharmacologist/epidemiologist Erasmus MC<br><br>**A.R. Dreijer MSc**, hospital pharmacist resident-PhD student Reinier de Graaf ziekenhuis
**Sponsor (in Dutch: verrichter/opdrachtgever)**	Erasmus MC<br>Postbus 2040<br>3000 CA ROTTERDAM<br><br>Reinier de Graaf ziekenhuis<br>Postbus 5011<br>2625 AD Delft

**Subsidizing party**	**DSW Zorgverzekeraar**<br>Postbus 173<br>3100 AD Schiedam<br><br>**Daiichi Sankyo**<br>Wilgenlaan 5<br>1161 JK Zwanenburg<br><br>**Boehringer Ingelheim**<br>Comeniusstraat 6<br>1817 MS Alkmaar<br><br>**Bayer Nederland**<br>Energieweg 1<br>Postbus 80<br>3641 RT Mijdrecht<br><br>**Pfizer Nederland**<br>Rivium Westlaan 142<br>2909LD Capelle a/d Ijssel
**Independent expert (s)**	**Dr. P.A.W. te Boekhorst**<br>Hematologist/transfusionspecialist<br>*T*: 010 703 31 33 | *E*: p.theboekhorst@erasmusmc.nl<br>Erasmus MC<br>Hematologie<br>Postbus 2040<br>3000 CA ROTTERDAM<br><br>**Drs. J.B. van den Bosch**<br>Anesthesiologist/intensivist<br>T: 015-2603385 | *E:* J.vandenBosch@rdgg.nl<br>Reinier de Graaf ziekenhuis<br>Intensive care<br>Postbus 5011<br>2625 AD Delft
**Laboratory sites**	**N.a.**
**Pharmacy**	**-**

PROTOCOL SIGNATURE SHEET

Name	Signature	Date
**Sponsor or legal representative:**<br><br>**Head of Department:**<br>- **Hospital pharmacy**	Dr. P.J. Roos, head of the department pharmacy Erasmus MC<br><br>Drs. E. Meijer, head of the department hospital pharmacy Reinier de Graaf ziekenhuis	20/5/15<br><br>21/5/15
**Coordinating Investigator/Project leader/Principal Investigator:**	Dr. P.M.L.A. van den Bemt, hospital pharmacist-clinical pharmacologist/epidemiologist	28/5/15

TABLE OF CONTENTS

1. INTRODUCTION AND RATIONALE 11
2. OBJECTIVES 13
3. STUDY DESIGN 14
4. STUDY POPULATION 15
4.1 Population (base) 15
4.2 Inclusion criteria 15
4.3 Exclusion criteria 15
4.4 Sample size calculation 15
5. TREATMENT OF SUBJECTS 17
5.1 Investigational product/treatment 17
5.2 Use of co-intervention 17
5.3 Escape medication 17
6. INVESTIGATIONAL PRODUCT 18
6.1 Name and description of investigational product(s) 18
6.2 Summary of findings from non-clinical studies 18
6.3 Summary of findings from clinical studies 18
6.4 Summary of known and potential risks and benefits 18
6.5 Description and justification of route of administration and dosage 18
6.6 Dosages, dosage modifications and method of administration 18
6.7 Preparation and labelling of Investigational Medicinal Product 18
6.8 Drug accountability 18
7. NON-INVESTIGATIONAL PRODUCT 19
7.1 Name and description of non-investigational product(s) 19
7.2 Summary of findings from non-clinical studies 19
7.3 Summary of findings from clinical studies 19
7.4 Summary of known and potential risks and benefits 19
7.5 Description and justification of route of administration and dosage 19
7.6 Dosages, dosage modifications and method of administration 19
7.7 Preparation and labelling of Non Investigational Medicinal Product 19
7.8 Drug accountability 19
8. METHODS 20
8.1 Study parameters/endpoints 20
8.1.1 Main study parameter/endpoint 20
8.1.2 Secondary study parameters/endpoints 20
8.1.3 Other study parameters 20
8.2 Randomization, blinding and treatment allocation 20
8.3 Study procedures 20
8.3.1 Pre-intervention phase 21
8.3.2 Implementation phase 28
8.3.3 Post-intervention phase 28
8.4 Withdrawal of individual subjects 28

8.4.1 Specific criteria for withdrawal 28
8.5 Replacement of individual subjects after withdrawal 28
8.6 Follow-up of subjects withdrawn from treatment 28
8.7 Premature termination of the study 28
9. SAFETY REPORTING 29
9.1 Section 10 WMO event 29
9.2 AEs, SAEs and SUSARs 29
9.2.1 Adverse events (AEs) 29
9.2.2 Serious adverse events (SAEs) 29
9.2.3 Suspected unexpected serious adverse reactions (SUSARs) 29
9.3 Annual safety report 29
9.4 Follow-up of adverse events 30
9.5 [Data Safety Monitoring Board (DSMB) / Safety Committee] 30
10. STATISTICAL ANALYSIS 31
10.1 Primary study parameter(s) 31
10.2 Secondary study parameter(s) 31
10.3 Other study parameters 31
10.4 Interim analysis (if applicable) 31
11. ETHICAL CONSIDERATIONS 32
11.1 Regulation statement 32
11.2 Recruitment and consent 32
11.3 Objection by minors or incapacitated subjects 32
11.4 Benefits and risks assessment, group relatedness 32
11.5 Compensation for injury 33
11.6 Incentives 33
12. ADMINISTRATIVE ASPECTS, MONITORING AND PUBLICATION 34
12.1 Handling and storage of data and documents 34
12.2 Monitoring and Quality Assurance 34
12.3 Amendments 34
12.4 Annual progress report 34
12.5 End of study report 34
12.6 Public disclosure and publication policy 34
13. STRUCTURED RISK ANALYSIS 35
13.1 Potential issues of concern 35
13.2 Synthesis 35
14. REFERENCES 36
ATTACHMENTS 38

LIST OF ABBREVIATIONS AND RELEVANT DEFINITIONS

**ABR**	**ABR form, General Assessment and Registration form, is the application form that is required for submission to the accredited Ethics Committee (In Dutch, ABR = Algemene Beoordeling en Registratie)**
**Anti-Xa**	**Antifactor Xa**
**ATC-code**	**Anatomical Therapeutic Chemical Classification System**
**A-TEAM**	**Antibiotic teams**
**AE**	**Adverse Event**
**APTT**	**Activated Partial Thromboplastin Time**
**AR**	**Adverse Reaction**
**CA**	**Competent Authority**
**CCMO**	**Central Committee on Research Involving Human Subjects; in Dutch: Centrale Commissie Mensgebonden Onderzoek**
**CV**	**Curriculum Vitae**
**DBC**	**Diagnose Behandeling Combinatie**
**DSMB**	**Data Safety Monitoring Board**
**DVT**	**Deep vein thrombosis**
**dTT**	**Diluted Thrombin Time**
**eGFR**	**Estimated Glomerular Filtration Rate**
**EU**	**European Union**
**EudraCT**	**European drug regulatory affairs Clinical Trials**
**EQ5D score**	**EuroQol EQ5D score**
**GCP**	**Good Clinical Practice**
**HARM**	**Hospital Admissions Related to Medication**
**Hb**	**Hemoglobin**
**HT**	**Hematocrit**
**IB**	**Investigator’s Brochure**
**IC**	**Informed Consent**
**IMP**	**Investigational Medicinal Product**
**IMPD**	**Investigational Medicinal Product Dossier**
**INR**	**International Normalized Ratio**
**ISTH**	**International Society on Thrombosis and Haemostasis**
**LOS**	**Length of the hospital stay**
**LSKA**	**Landelijke Standaard Ketenzorg Antistolling**
**MARS**	**Medication Adherence Rating Scale**
**METC**	**Medical research ethics committee (MREC); in Dutch: Medisch Ethische Toetsing Commissie (METC)**
**NOACs**	**New oral anticoagulants**
**PE**	**Pulmonary embolism**
**PT**	**Prothrombin time**
**(S)AE**	**(Serious) Adverse Event**
**SPC**	**Summary of Product Characteristics (in Dutch: officiële productinfomatie IB1-tekst)**

**Sponsor**	**The sponsor is the party that commissions the organisation or performance of the research, for example a pharmaceutical company, academic hospital, scientific organisation or investigator. A party that provides funding for a study but does not commission it is not regarded as the sponsor, but referred to as a subsidising party.**
**S-TEAM**	**Stollingsteam (antithrombotic team)**
**SUSAR**	**Suspected Unexpected Serious Adverse Reaction**
**TTR**	**Time in therapeutic range**
**VKA**	**Vitamin K antagonists**
**VTE**	**Venous thromboembolism**
**Wbp**	**Personal Data Protection Act (in Dutch: Wet Bescherming Persoonsgevens)**
**WMO**	**Medical Research Involving Human Subjects Act (in Dutch: Wet Medisch-wetenschappelijk Onderzoek met Mensen**

SUMMARY

Rationale: Antithrombotic therapy carries high risks for patient safety. The Dutch HARM study showed that antithrombotics belong to the top 5 of medication involved in potentially preventable HARMs. Multidisciplinary teams have been proposed to improve the safety of antithrombotic therapy. However, most antithrombotic services are described mainly as pharmacist-led antithrombotic services in US hospitals that are predominantly aimed at warfarin dosing, which differs from the Dutch situation. At present the effect of a multidisciplinary antithrombotic team in the Dutch situation is unknown. The hypothesis is that the introduction of a multidisciplinary antithrombotic team (in Dutch 'Stollingsteam' or S-team) in hospital will improve the efficacy and safety of antithrombotic therapy.
Objective: The main objective of this study is to determine the effect of a multidisciplinary antithrombotic team ('S-team') on the frequency of a composite endpoint consisting of bleeding and thrombotic events. The secondary objectives are to study the effect of a S-team on the frequency of bleeding events, frequency of thrombotic events, severity of bleeding complications, length of hospital stay, readmissions, quality of life, adherence by the patient to the therapy, quality of care (patient satisfaction with antithrombotic therapy), adherence by the doctors to the hospital protocol, healthcare costs, all-cause mortality and percent time in therapeutic range of vitamin K antagonists.
Study design: An intervention study with a before- and after design.
Study population: Patients that are or will be treated with antithrombotics during admission to the university medical center Erasmus MC Rotterdam or general teaching hospital Reinier de Graaf Ziekenhuis Delft are included in the study. We aim to include 1834 patients, 917 patients in the pre-intervention phase and 917 patients in the post-intervention phase.
Intervention: The intervention consists of the implementation of the S-team.
Main study parameters/endpoints: Proportion of patients with a composite endpoint consisting of ≥1 bleeding (major bleeding and mild to moderate clinically relevant non-major bleeding) or ≥1 thrombotic event from time to start with antithrombotic medication (or hospitalization) until 3 months after hospitalization.
Nature and extent of the burden and risks associated with participation, benefit and group relatedness: At discharge the patient will be asked to fill out a consent form. By signing the consent form, the patient gives permission to retrieve patient data from the community pharmacist and the general practitioner. In addition, the patient receives four small questionnaires 3 months after hospitalization. It will take about 20 minutes to fill out the questionnaires.

1. INTRODUCTION AND RATIONALE

Antithrombotic therapy carries high risks for patient safety, as was clearly shown in the Dutch HARM - (Hospital Admissions Related to Medication) study and subsequent reports ('Monitor Zorggerelateerde Schade', from 2004, 2008 and 2012)[1]. The Dutch HARM study showed that antithrombotics belong to the top 5 of medication involved in potentially preventable HARMs.

In response to the HARM study, a multidisciplinary guideline was drafted to provide a standard for antithrombotic therapy and stress the importance of providing optimal care to patients on antithrombotic therapy: the 'Landelijke Standaard Ketenzorg Antistolling' (LSKA)[2].

The mere publication of the LSKA does not guarantee its implementation. A parallel can be drawn with active policy on reduction of antibiotic resistance: all hospitals are involved in such policies, but still only recently the introduction of antibiotic stewardship was proposed in order to further enhance such policies. Multidisciplinary antibiotic teams (A-teams) are considered useful for optimization of therapy[3]. Antithrombotics obviously carry more risk than antibiotics (bleeding and thrombotic complications), so it is logical to presume that multidisciplinary teams (in Dutch 'Stollingsteams' or S-teams) focusing on antithrombotics will be even more useful.

With non-vitamin K oral anticoagulants (NOACs) being marketed, it is unclear who will become responsible for optimization of their use. As vitamin K antagonists (VKAs) are the responsibility of Thrombosis Services, doctors are no longer used to monitor antithrombotic therapy, adherence and drug-drug interactions. Therefore, the supervision of therapy with NOACs may very well be insufficiently attended to. A solution would be to expand the work processes of the Thrombosis Services, but evidence on the effect of such expanded services is mostly lacking. Furthermore, with patients being hospitalized for increasingly shorter periods, optimization of transfer of anticoagulation therapy after discharge becomes of paramount importance.

Studies on the effect of a multidisciplinary antithrombotic team are scarce. Antithrombotic services are described mainly as pharmacist-led antithrombotic services in US hospitals that are predominantly aimed at warfarin dosing[4]. This differs from the Dutch situation, where Thrombosis Services with a medical leader are responsible. In only 4 of 25 respondents of a survey such an antithrombotic service was multidisciplinary[4]. One article describes an expanded antithrombotic stewardship, including both NOAC treatment and facilitating care after hospital discharge[5]. It concerned a US one center pharmacist-led stewardship and the study included only a small retrospective control group of 12 patients. The majority of results were described for 409 patients monitored after implementation of the program. Also, the transition after discharge included only 34 patients, with 30 of them on warfarin therapy. Interventions consisted of changes to a more appropriate antithrombotic according to guidelines and dosing corrections. The length of hospital stay was reduced by 1.5 days and cost savings were $270320 in 1.5 years[5]. Discharge patient education and knowledge of patients is described in a few studies, but again mostly on warfarin[6,7]. Given the known effect of patient education on adherence, patient education will be much more important for the NOAC therapy in which monitoring cannot be used to verify adherence.

The (lack of) implementation of the LSKA, and the shifting responsibilities regarding NOACs need solutions aiming at safe use of antithrombotics. The intervention in the present study consists of the implementation of an S-team. An S-team can be made responsible for LSKA implementation, can act as an expert team that can be consulted both from in- and outside the hospital, can take care of adequate handovers of patients discharged from hospital, and can improve patient education. A properly informed patient will more likely use antithrombotics safely and adherently, resulting in less complications.

At present the effectiveness of a multidisciplinary antithrombotic team is unknown. Therefore, the main objective of this study is to determine the effect of a multidisciplinary antithrombotic team ('S-team') on the frequency of bleeding and thrombotic events in two Dutch hospitals.

2. OBJECTIVES

Primary Objective:

To determine the effect of a multidisciplinary antithrombotic team on the frequency of a composite endpoint consisting of bleeding and thrombotic events.

Secondary Objective(s):

The following secondary objectives when a multidisciplinary antithrombotic team (S-team) is installed in the hospital are:

1. To determine the effect on frequency of bleeding events.
2. To determine the effect on frequency of thrombotic events.
3. To determine the effect on severity of bleeding complications.
4. To determine the effect on length of the hospital stay.
5. To determine the effect on percentage of (re)admissions caused by antithrombotic medication.
6. To determine the effect on quality of life, adherence by the patient to the therapy and quality of care (patient satisfaction with antithrombotic therapy).
7. To determine the effect on adherence to the hospital protocol.
8. To determine the effect on costs.
9. To determine the effect on all-cause mortality.
10. To determine the effect on percent time in therapeutic range of vitamin K antagonists.

3. STUDY DESIGN

This is an intervention study with a before- and after design, with the intervention being a quality improvement as is mandated by the national guideline LSKA[2].

4. STUDY POPULATION

4.1 Population (base)

The base population is formed by patients that are or will be treated with antithrombotics admitted to the university medical center Erasmus MC Rotterdam and general teaching hospital Reinier de Graaf Ziekenhuis Delft.

The Erasmus MC is a 1320-bed university medical center based in Rotterdam, Netherlands. Het Reinier de Graaf Ziekenhuis is a general teaching hospital located in Delft, Netherlands and has 590 beds.

The study is carried out from 2015 to 2018.

4.2 Inclusion criteria

In order to be eligible to participate in this study, a subject must meet all of the following criteria:

- Patients that are or will be treated with antithrombotics in the Erasmus MC or in the Reinier de Graaf hospital.
- Informed consent (we will ask permission from the patients to collect community pharmacy and to fill out four questionnaires).
- Only the first hospital admission of a patient within the study period will be included (readmission is an endpoint).

4.3 Exclusion criteria

A potential subject who meets any of the following criteria will be excluded from participation in this study:

- No informed consent
- Patients admitted for one day
- Patients admitted to the Intensive Care Unit
- Patients treated with low molecular weight heparins monotherapy for thrombosis prophylaxis
- Patients enrolled in a clinical trial of antithrombotic therapy
- Patients treated with phytomenadione used for the prevention and treatment of vitamin K deficiency
- Patients treated during hospitalization with a single dose of the antithrombotic agents

4.4 Sample size calculation

In studies major bleeding complications tend to occur in about 2-3% of all patients per year on antithrombotics[8,9], but in every day practice it seems this rate is at least 10%[10]. Conservatively, we will presume a rate of 5%. Thrombotic events are estimated to occur in 3% of treated patients per year in studies[9]. As trials form the ideal situation, we assume this is the rate we could achieve with the S-team and that the rate is 4% in the pre-intervention period. This results in a composite rate of 9% and we expect to decrease this to 2.5% bleeding events and 3% thrombotic events (composite 5.5%). With an alpha of 0.05 and power of 0.80 the required sample size will be 917 patients in the pre-intervention phase and 917 patients in the post-intervention phase. In order to account for drop-outs 1900 patients will be included.

In a (not yet published) study aiming at the development of a prediction rule for VKA-related International Normalized Ratio (INR) elevation from our Erasmus MC group 3800 patients using VKAs were included over 4 years. Thus within 9 months over 700 patients using VKAs will be admitted to the Erasmus MC alone. As we include another hospital and other antithrombotics as well, we expect that 9 months will be sufficient to recruit the required number of patients.

5. TREATMENT OF SUBJECTS

5.1 Investigational product/treatment

The intervention consists of the implementation of the S-team, which is a quality improvement dictated by the national guideline LSKA. The S-team will consist of a specialized thrombosis nurse as case manager, hematologist, medical leader Thrombosis Service, hospital pharmacist/clinical pharmacologist, cardiologist and a pediatric hematologist. An anesthesiologist, neurologist, pulmonologist, dermatologist, clinical chemist, emergency physician and (orthopedic) surgeon may be added to the team when necessary.

The main tasks of the S-team are:

- Drafting of local guidelines
- Monitoring of the medication by the hospital pharmacist/clinical pharmacologist focused on patients that are treated with antithrombotics
- Patient instructions
- Proper handover to either the Thrombosis Service or the general practitioner, and to the community pharmacist, at the moment of hospital discharge
- Educating physicians, nurses and hospital pharmacists
- Offering consultation for professionals in- and outside the hospital

5.2 Use of co-intervention

None.

5.3 Escape medication

N.a.

6. INVESTIGATIONAL PRODUCT

6.1 Name and description of investigational product(s)
N.a.

6.2 Summary of findings from non-clinical studies
N.a.

6.3 Summary of findings from clinical studies
N.a.

6.4 Summary of known and potential risks and benefits
N.a.

6.5 Description and justification of route of administration and dosage
N.a.

6.6 Dosages, dosage modifications and method of administration
N.a.

6.7 Preparation and labelling of Investigational Medicinal Product
N.a.

6.8 Drug accountability
N.a.

7. NON-INVESTIGATIONAL PRODUCT

7.1 Name and description of non-investigational product(s)
N.a.

7.2 Summary of findings from non-clinical studies
N.a.

7.3 Summary of findings from clinical studies
N.a.

7.4 Summary of known and potential risks and benefits
N.a.

7.5 Description and justification of route of administration and dosage
N.a.

7.6 Dosages, dosage modifications and method of administration
N.a.

7.7 Preparation and labelling of Non Investigational Medicinal Product
N.a.

7.8 Drug accountability
N.a.

8. METHODS

8.1 Study parameters/endpoints

8.1.1 Main study parameter/endpoint

Composite endpoint: proportion of patients with ≥1 bleeding (major bleeding and mild to moderate clinically relevant non-major bleeding) or ≥1 thrombotic events from time to start with antithrombotic medication (or hospitalization) until 3 months after hospitalization.

8.1.2 Secondary study parameters/endpoints

- Proportion of patients with ≥1 bleeding (major bleeding and mild to moderate clinically relevant non-major bleeding) event from time to start with antithrombotic medication (or hospitalization) until 3 months after hospitalization.
- Proportion of patients with ≥1 thrombotic event from time to start with antithrombotic medication (or hospitalization) until 3 months after hospitalization.
- Severity of bleeding complication
- Length of hospital stay (LOS)
- Percentage of readmissions within 3 months after discharge
- Patient related outcomes (quality of life, adherence of patient to therapy and patient satisfaction with the quality of care of the antithrombotic therapy)
- Adherence to the hospital protocol
- Healthcare costs (labor costs versus costs for bleeding/thrombotic events (including use of antidotes))
- All-cause mortality
- Percent time in therapeutic range of vitamin K antagonists during hospitalization and 3 months after discharge.

8.1.3 Other study parameters

N.a.

8.2 Randomization, blinding and treatment allocation

N.a.

8.3 Study procedures

The study consists of three phases: the pre-intervention phase, the implementation phase and the post-intervention phase (figure 1).

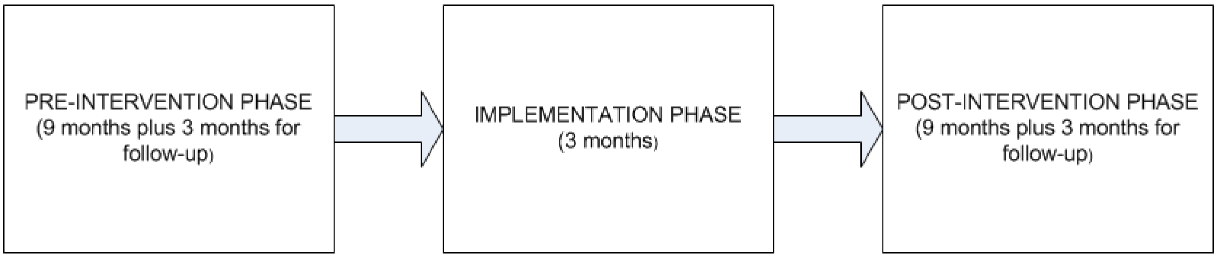

Figure 1: Phases during the study

8.3.1 Pre-intervention phase

During a 9-month period usual care is provided to patients. This means that antithrombotic therapy, related patient instructions and handover after discharge are carried out by the physicians responsible for the patient. Patients that are or will be treated with antithrombotics in the Erasmus MC and in the Reinier de Graaf hospital will be included with the help of a clinical rule. The criteria on which the clinical rule screens are the use of antithrombotic drugs which are listed in table 1 and the use of antidotes which are listed in table 2.

Table 1: Antithrombotic agents

**Group of antithrombotic agents (ATC code)**	**Antithrombotic agents (ATC code)**
Vitamin K antagonists (B01AA)	Phenprocoumon (B01AA04)
	Acenoucoumarol (B01AA07)
Heparin group (B01AB)	Heparin (B01AB01)
	Antithrombin III (B01AB02)
	Dalteparin (B01AB04)
	Enoxaparin (B01AB05)
	Nadroparin (B01AB06)
	Danaparoid (B01AB09)
	Tinzaparin (B01AB10)
Direct thrombin inhibitors (B01AE)	Bivaluridin (B01AE06)
	Dabigatran etexilate (B01AE07)
Direct factor Xa inhibitors (B01AF)	Rivaroxaban (B01AF01)
	Apixaban (B01AF02)
Other antithrombotic agents (B01AX)	Fondaparinux (B01AX05)

Table 2: Antidotes

**Group of antihemorrhagics (ATC code)**	**Antihemorrhagics (ATC code)**
Amino acids (B02AA)	Tranexamic acid (B02AA02)
Vitamin K (B02BA)	Phytomenadione (B02BA01)
Fibrinogen (B02BB)	Fibrinogen, human (B02BB01)
Blood coagulation factors (B02BD)	Coagulation factor IX, II, VII and X in combination (B02BD01)
	Eptacog alfa (activated) (B02BD08)
Antidotes (V03AB)	Protamine (V03AB14)

New antithrombotic agents and antihemorrhagics entering the market, are also included in the study when they are introduced in the hospital.

During the hospitalization for each included patient data are collected in a case report form (CRF). Detailed information on the data can be found in table 3.

Table 3: Content of case report (CRF)

**Part**	**Data content**
Patient data	Patient ID
	Date of birth*
	Gender*
	Weight on the first day of hospitalization*
	Length*
	Community pharmacist*[$]
	Reason for hospitalization*
	Reason for exclusion
	(Co)morbidity*
	Day of hospitalization*
	Any surgery (coded with Verrichtingen code[11]) or diagnosis during hospitalization*
Study outcomes	Bleeding (major bleeding and mild to moderate clinical relevant non-major bleeding) or thrombotic event during hospitalization*
	Bleeding (major bleeding and mild to moderate clinical relevant non-major bleeding) or thrombotic event within 3 months after hospitalization[^]
	Severity of bleeding complication*
	Hospital discharge date*
	Date of each readmission in the following 3 months after the first hospitalization*[^]
	The reason for readmission[^]
	Quality of life[12-14] (3 months after discharge):[$]<br>- Age 0-3: no EQ-5D-Y available<br>- Age 4-7: EQ-5D-Yproxy version 1<br>- Age 8-11: EQ-5D-Y or EQ-5D-Y proxy version 1<br>- Age 12-15: EQ-5D-Y or EQ5D<br>- Age 16 and older: EQ5D
	Adherence by the patient to the therapy; MARS5[15-18] (3 months after discharge)[$]
	Patient satisfaction about the quality of care of the antithrombotic therapy; VAS satisfaction scale[19] (3 months after discharge)[$]
	Adherence to the hospital protocol

	Percent time in therapeutic range (TTR) of vitamin K antagonists during hospitalization and as an outpatient during 3 months follow-up*
	All-cause mortality*[+]
Clinical data	Laboratory values and the date of determination*[+]<br>- International Normalized Ratio (INR)<br>- Activated Partial Thromboplastin Time (APTT)<br>- Prothrombin time (PT)<br>- Diluted Thrombin Time (dTT)<br>- Hemoglobin (Hb)<br>- Antifactor Xa (Anti-Xa)<br>- Creatinine<br>- Hematocrit (HT)<br>- Erythrocytes<br>- Thrombocytes<br>- Estimated Glomerular Filtration Rate (eGFR)<br>- Weight<br>(Available clinical data is collected from 3 months before inclusion till 3 months after hospitalization)
Medication data	Medication use during hospitalization (coded with ATC-code[20])*
	Use of antidotes: vitamin K, protamine sulfate, prothrombin complex concentrate, fibrinogen, tranexamic acid and recombinant factor VIIa (coded with ATC code[19])*
	Use of blood products: blood transfusion and other blood products*
	Overview of medication use three months before hospitalization (coded with ATC-code[20])[+]
	Overview of medication use three months after hospitalization (coded with ATC-code[20])[+]

*Obtained from medical record of the hospital information system
[+]Obtained from the community pharmacist and the Thrombosis Service
[$]Obtained from the patient using questionnaire
^Obtained by sending a small questionnaire asking for visits to the general practitioner or hospital because of a bleeding or thrombotic event within 3 months after hospitalization.

The primary outcome of the study is the proportion of patients with a composite endpoint consisting of ≥1 bleeding (major bleeding and mild to moderate clinical relevant non-major bleeding) or ≥1 thrombotic events during and 3 months after hospitalization.

Patients treated with antithrombotics and/or antidotes in the Erasmus MC and in the Reinier de Graaf hospital will be included with the help of a clinical rule (table 1 and 2). The data from the medical record of the hospital information system is used to determine whether there is a bleeding (major bleeding and mild to moderate clinical relevant non-major bleeding) or thrombotic event in patients who are enrolled in the study.

After hospitalization data from community pharmacist, general practitioner and the Thrombosis Services are used. At discharge the patient will be asked to fill in a consent form. By signing the consent form, the patient gives permission to retrieve patient data from the community pharmacist, general practitioner and the Thrombosis Services.

Definition of terms:

The ISTH definitions of bleeding in patients are as follows:

- Major Bleeding in non-Surgical Patients[21]
  - 1. Fatal bleeding, and/or
  - 2. Symptomatic bleeding in a critical area or organ, such as intracranial, intraspinal, intraocular, retroperitoneal, intra-articular or pericardial, or intramuscular with compartment syndrome, and/or
  - 3. Bleeding causing a fall in hemoglobin level of 20 g L^{-1} (1.24 mmol L^{-1}) or more, or leading to transfusion of two or more units of whole blood or red cells.
- Major bleeding in surgical patients[22]
  - 1. Fatal bleeding, and/or
  - 2. Bleeding that is symptomatic and occurs in a critical area or organ, such as intracranial, intraspinal, intraocular, retroperitoneal, pericardial, in a non-operated joint, or intramuscular with compartment syndrome, assessed in consultation with the surgeon, and/or
  - 3. Extrasurgical site bleeding causing a fall in hemoglobin level of 20 g L^{-1} (1.24 mmol L^{-1}) or more, or leading to transfusion of two or more units of whole blood or red cells, with temporal association within 24–48 h to the bleeding, and/or
  - 4. Surgical site bleeding that requires a second intervention (open arthroscopic, endovascular) or a hemarthrosis of sufficient size as to interfere with rehabilitation by delaying mobilization or delayed wound healing, resulting in prolonged hospitalization or a deep wound infection, and/or
  - 5. Surgical site bleeding that is unexpected and prolonged and/or sufficiently large to cause hemodynamic instability, as assessed by the surgeon. There should be an associate fall in hemoglobin level of at least 20 g L^{-1} (1.24 mmol L^{-1}), or transfusion, indicated by the bleeding, of at least two units of whole blood or red cells, with temporal association within 24 h to the bleeding.

- Mild to moderate bleeding:
    - All bleeding events that do not meet the International Society of Thrombosis and Haemostasis (ISTH)[21] criteria according to which major bleeding is defined.

Thrombotic event: any arterial or venous thrombosis, including acute myocardial infarction or stroke for arterial thrombosis and deep venous thrombosis or pulmonary embolism for venous thrombosis.

Definition of terms:

- Acute myocardial infarction: Detection of a rise and/or fall of cardiac biomarker values [preferably cardiac troponin (cTn)] with at least one value above the 99th percentile upper reference limit (URL) and with at least one of the following[23]:
    - Symptoms of ischemia
    - New or presumed new significant ST-segment–T wave (ST–T) changes or new left bundle branch block (LBBB).
    - Development of pathological Q waves in the ECG.
    - Imaging evidence of new loss of viable myocardium or new regional wall motion abnormality.
    - Identification of an intracoronary thrombus by angiography or autopsy.
- Stroke: an embolic, thrombotic, or haemorrhagic vascular event or stroke with motor, sensory, or cognitive dysfunction (such as hemiplegia, hemiparesis, aphasia, sensory deficit, impaired memory) that persisted for 24 or more hours.[24]
- Deep venous thrombosis: venous thromboembolism is defined as an acute vascular occlusion of an extremity or organ, documented by means of imaging, surgery, or autopsy.[25]
- Pulmonary embolism: the presence of a blood clot in a pulmonary artery with subsequent obstruction of blood supply to the lung parenchyma or if the patient had a ventilation-perfusion scan interpreted as high probability of pulmonary embolism or a positive result on spiral computed tomography, transesophageal echocardiography, pulmonary arteriography, or computed tomography angiography.[26]
- Any objectively determined venous thrombus

The secondary outcomes are severity of bleeding complication, length of hospital stay, readmissions within 3 months after discharge, quality of life, quality of care (patient satisfaction with antithrombotic therapy), adherence by the patient to the therapy, healthcare costs, adherence by the doctors to the hospital protocol, all-cause mortality and percent time in therapeutic range of vitamin K antagonists.

Severity of bleeding complication

The ISTH/SSC proposed a bleeding assessment tool that facilitates the diagnosis of less symptomatic mild bleeding disorder and the grading of severity in patients with known inherited bleeding disorders. For each specific bleeding symptom, a score of 1 or more is classified as significant.[27]

Readmissions within 3 months after discharge

Three months after hospitalization the patient receives a small questionnaire about the use of healthcare. The questionnaire asks for visits to the general practitioner or the hospital because of a bleeding or thrombotic event within 3 months after hospitalization.

The hospital information system is used to register readmissions and the reason for readmission of the patients in the same hospital. The patient’s general practitioner is asked for readmissions in other hospitals. Only the patient’s first hospital admission is included in the study period (readmissions are the secondary outcome measures).

It will take about 5 minutes to fill out the questionnaire.

Quality of life[12-14]

At discharge the patient (or the parents/guardian of the patient) will be asked to fill out a consent form. After the patient (or the parents/guardian of the patient) has signed the consent form, he or she receives a EuroQol EQ5D questionnaire 3 months after hospitalization. The questionnaire will ask for the quality of life. The age range of patients of the quality of life version is as follows:

- Age 0-3: no EQ-5D-Y is available.
- Age 4-7: EQ-5D-Y proxy version 1: the proxy rates how he/she rates the health of the child.
- Age 8-11: EQ-5D-Y or EQ-5D-Y proxy version 1: Generally EQ-5D-Y is recommended. However, EQ-5D-Y proxy version 1 is usable for children of 8+ who are not able to fill in the EQ-5D-Y themselves.
- Age 12-15: EQ-5D-Y or EQ5D: Generally EQ-5D-Y is recommended.
- Age 16 and older: EQ5D

It will take about 5-10 minutes to fill out the questionnaire.

Adherence by the patient to the therapy[15-18]

At discharge the patient (or the parents/guardian of the patient) will be asked to fill out a consent form. After the patients (or the parents/guardian of the patient) has signed the consent form they are asked 3 months after hospitalization to fill out a questionnaire about their adherence to drug treatment (MARS; Medication Adherence Rating Scale). Adherence to medication is assessed using a five-item self-report scale called the Medication Adherence Report Scale 5 (MARS5[16]). The MARS5 contains five items to which respondents rate their

agreement on a five-point scale. Scores for each of the five items are summed to give a total adherence score between 5 and 25, where higher scores indicate higher levels of adherence. The age range of patients is as follows:
- Age 0-8: the questionnaire is filled out by the parents/guardian of the patients.
- Age ≥9: the questionnaire is filled out by the patient himself. (A child's ability to respond to the questionnaire was determined by his/her physician)[28].

It will take about 5 minutes to fill out the questionnaire.

Patient satisfaction with the quality of care of the antithrombotic therapy[19]
At discharge the patient (or the parents/guardian of the patient) will be asked to fill out a consent form. After the patient (or the parents/guardian of the patient) has signed the consent form, he or she receives a VAS satisfaction scale 3 months after hospitalization. The questionnaire will ask for the satisfaction with the quality of care.

It will take less than 1 minute to fill out the questionnaire.

Bleeding (major bleeding and mild to moderate clinical relevant non-major bleeding) or thrombotic event within 3 months after hospitalization
Three months after hospitalization the patient receives a small questionnaire about the use of healthcare. One of the questions is whether the patient visited the general practitioner or the hospital because of a bleeding or thrombotic event within 3 months after hospitalization.

Adherence to the hospital protocol
Information from the medical record of the hospital information system will be used to verify the adherence by the doctors to the antithrombotic hospital protocol.

Cost-effectiveness analysis
The aim of the economic evaluation is to determine whether the implementation of the S-team contributes to a reduction of the healthcare costs. All costs of healthcare are assessed as it is hard to distinguish which costs are related to medication use. Healthcare costs include the costs of hospitalizations for bleeding/thrombotic events, labor costs and medication costs (including the use of antidotes).

Medication use three months before and three months after hospitalization
Medication records of the community pharmacy can be consulted through a link in the hospital information system for patients that are within the catchment area of the hospital. If a community pharmacist is not connected to the hospital information system, the hospital pharmacy will obtain a faxed medication list from the community pharmacist.

All-cause mortality
We define all-cause mortality as death from any cause during and 3 months after hospitalization.

Percent time in therapeutic range of vitamin K antagonists
INR data are collected during and 3 months after hospitalization. Therapeutic INR range (TTR) is a way of summarizing INR control over time.

8.3.2 Implementation phase

The intervention consists of the implementation of the S-team. The S-team consisting of a specialized thrombosis nurse as case manager, hematologist, medical leader Thrombosis Service, hospital pharmacist/clinical pharmacologist, cardiologist and a pediatric hematologist. An anesthesiologist, neurologist, pulmonologist, dermatologist, clinical chemist, emergency physician and (orthopedic) surgeon may be added to the team when necessary.

8.3.3 Post-intervention phase

During a 9-month period the S-team is active. From the clinical rule patients with antithrombotics will be identified and presented to the S-team. The main tasks of the S-team will be:

- Drafting of local guidelines
- Monitoring of the medication by the hospital pharmacist/clinical pharmacologist focused on patients that are treated with antithrombotics
- Patient instructions
- Proper handover to either the Thrombosis Service or the general practitioner, and to the community pharmacist, at the moment of hospital discharge
- Educating physicians, nurses and hospital pharmacists
- Offering consultation for professionals in- and outside the hospital

During the post-intervention phase the same data are collected as during the pre-intervention phase.

8.4 Withdrawal of individual subjects

N.a.

8.4.1 Specific criteria for withdrawal

N.a.

8.5 Replacement of individual subjects after withdrawal

N.a.

8.6 Follow-up of subjects withdrawn from treatment

N.a.

8.7 Premature termination of the study

N.a.

9. SAFETY REPORTING

9.1 Section 10 WMO event

In accordance to section 10, subsection 1, of the WMO, the investigator will inform the subjects and the reviewing accredited METC if anything occurs, on the basis of which it appears that the disadvantages of participation may be significantly greater than was foreseen in the research proposal. The study will be suspended pending further review by the accredited METC, except insofar as suspension would jeopardize the subjects' health. The investigator will take care that all subjects are kept informed.

9.2 AEs, SAEs and SUSARs

9.2.1 Adverse events (AEs)

Adverse events are defined as any undesirable experience occurring to a subject during the study, whether or not considered related to the implementation of the S-team. All adverse events reported spontaneously by the subject or observed by the investigator or his staff will be recorded.

9.2.2 Serious adverse events (SAEs)

A serious adverse event is any untoward medical occurrence or effect that at any dose:

- results in death;
- is life threatening (at the time of the event);
- requires hospitalization or prolongation of existing inpatients' hospitalization;
- results in persistent or significant disability or incapacity;
- is a congenital anomaly or birth defect;
- any other important medical event that may not result in death, be life threatening, or require hospitalization, may be considered a serious adverse experience when, based upon appropriate medical judgment, the event may jeopardize the subject or may require an intervention to prevent one of the outcomes listed above.

The sponsor will report the SAEs through the web portal *ToetsingOnline* to the accredited METC that approved the protocol, within 15 days after the sponsor has first knowledge of the serious adverse events.

SAEs that result in death or are life threatening should be reported expedited. The expedited reporting will occur not later than 7 days after the responsible investigator has first knowledge of the adverse event. This is for a preliminary report with another 8 days for completion of the report.

This section is not applicable for this quality project

9.2.3 Suspected unexpected serious adverse reactions (SUSARs)

N.a.

9.3 Annual safety report

N.a.

9.4 Follow-up of adverse events

All AEs will be followed until they have abated, or until a stable situation has been reached. Depending on the event, follow up may require additional tests or medical procedures as indicated, and/or referral to the general physician or a medical specialist. SAEs need to be reported till end of study within the Netherlands, as defined in the protocol

9.5 [Data Safety Monitoring Board (DSMB) / Safety Committee]

N.a.

10. STATISTICAL ANALYSIS

10.1 Primary study parameter(s)

The database will be made in Open Clinica (open source clinical trial software for Electronic Data Capture (EDC) Clinical Data Management (CDM)) and the results will be analyzed with a statistical analysis software program (SPSS).

For the primary outcome interrupted time series analysis will be used for data-analysis. The primary outcome will be compared using univariate and multivariate logistic regression analysis.

10.2 Secondary study parameter(s)

For the secondary outcomes linear or logistic regression analysis will be used, depending on the parameter.

To assess differences in patient characteristics for the two periods the appropriate test (t-test, Mann-Whitney U test or Pearson's chi-square test) will be used.

10.3 Other study parameters

N.a.

10.4 Interim analysis (if applicable)

N.a.

11. ETHICAL CONSIDERATIONS

11.1 Regulation statement

This study will be conducted according to the principles of the Declaration of Helsinki (version October, 2013) and in accordance with the Medical Research Involving Human Subjects Act (WMO).

11.2 Recruitment and consent

Pre-intervention phase:
At discharge the patient (or the parents/guardian of the patient) will be asked to fill out a consent form. By signing the consent form, the patient (or the parents/guardian of the patient) gives permission to retrieve patient data from the community pharmacist and the general practitioner.

After the patient (or the parents/guardian of the patient) has signed the consent form, he or she receives four small questionnaires 3 months after hospitalization. The questionnaires concern the quality of life, the quality of care (patient satisfaction with antithrombotic therapy), the adherence to drug treatment and the use of healthcare. It will take about 15-20 minutes to fill out the questionnaires.

If the four questionnaires are not returned within two weeks, we will contact the patient by telephone. The questionnaires will then be conducted by telephone.

Post-intervention phase:
At discharge the patient (or the parents/guardian of the patient) will be asked to fill out a consent form. By signing the consent form, the patient (or the parents/guardian of the patient) gives permission to retrieve patient data from the community pharmacist and the general practitioner.

The questionnaires concern the quality of life, the quality of care (patient satisfaction with antithrombotic therapy), the adherence to drug treatment and the use of healthcare. It will take about 15-20 minutes to fill out the questionnaires.

If the four questionnaires are not returned within two weeks, we will contact the patient by telephone. The questionnaires will then be conducted by telephone.

11.3 Objection by minors or incapacitated subjects

In case children object to participation, they will be withdrawn from the study.

11.4 Benefits and risks assessment, group relatedness

The study is an evaluation of a quality improvement measure. The benefit of this quality project may be the increase of the safety in patients who are using antithrombotic therapy. The study will take a total of approximately 15-20 minutes (time that represents the completion of the questionnaires) for the patient; all other data are collected by retrospective medical record screening. There are no components in this study that are related with possible adverse effects.

11.5 Compensation for injury

The sponsor/investigator has a liability insurance which is in accordance with article 7, subsection 9 of the WMO.

The sponsor (also) has an insurance which is in accordance with the legal requirements in the Netherlands (Article 7 WMO and the Measure regarding Compulsory Insurance for Clinical Research in Humans of 23th June 2003). This insurance provides cover for damage to research subjects through injury or death caused by the study.

1. € 450.000,-- (i.e. four hundred and fifty thousand Euro) for death or injury for each subject who participates in the Research;
2. € 3.500.000,-- (i.e. three million five hundred thousand Euro) for death or injury for all subjects who participate in the Research;
3. € 5.000.000,-- (i.e. five million Euro) for the total damage incurred by the organisation for all damage disclosed by scientific research for the Sponsor as ‘verrichter’ in the meaning of said Act in each year of insurance coverage.

The insurance applies to the damage that becomes apparent during the study or within 4 years after the end of the study.

11.6 Incentives

N.a.

12. ADMINISTRATIVE ASPECTS, MONITORING AND PUBLICATION

12.1 Handling and storage of data and documents

Data will be recorded and reported according to Good Clinical Practice. Data on subjects collected in the course of this trial will be documented in an anonymous fashion, i.e. the subject will be identified only by a subject number. Should knowledge of subject identity become necessary for safety or regulatory reasons, confidentiality by the investigators will be maintained.

All data will be kept in the trial archive for 15 years.

12.2 Monitoring and Quality Assurance

N.a.

12.3 Amendments

Amendments are changes made to the research after a favourable opinion by the accredited METC has been given. All amendments will be notified to the METC that gave a favourable opinion.

12.4 Annual progress report

The sponsor/investigator will submit a summary of the progress of the trial to the accredited METC once a year. Information will be provided on the date of inclusion of the first subject, numbers of subjects included and numbers of subjects that have completed the trial, serious adverse events/ serious adverse reactions, other problems, and amendments.

12.5 End of study report

The investigator will notify the accredited METC of the end of the study within a period of 8 weeks. The end of the study is defined as the last patient's last visit.

In case the study is ended prematurely, the investigator will notify the accredited METC within 15 days, including the reasons for the premature termination.

Within one year after the end of the study, the investigator/sponsor will submit a final study report with the results of the study, including any publications/abstracts of the study, to the accredited METC.

12.6 Public disclosure and publication policy

The results of the study will be presented in a peer reviewed publication.

13. STRUCTURED RISK ANALYSIS

13.1 Potential issues of concern

There are no issues of concern in this study because the intervention is a quality improvement as is mandated by the national guideline LSKA.

a. Level of knowledge about mechanism of action
N.a.

b. Previous exposure of human beings with the test product(s) and/or products with a similar biological mechanism
N.a.

c. Can the primary or secondary mechanism be induced in animals and/or in *ex-vivo* human cell material?
N.a.

d. Selectivity of the mechanism to target tissue in animals and/or human beings
N.a.

e. Analysis of potential effect
N.a.

f. Pharmacokinetic considerations
N.a.

g. Study population
N.a.

h. Interaction with other products
N.a.

i. Predictability of effect
N.a.

j. Can effects be managed?
N.a.

13.2 Synthesis

N.a.

14. REFERENCES

1. Leendertse AJ, Egberts AC, Stoker LJ, van den Bemt PM, HARM Study Group. Frequency of and risk factors for preventable medication-related hospital admissions in the Netherlands. Arch Intern Med 2008;168:1890-6.
2. Landelijke Standaard Ketenzorg Antistolling voor de eerste- en tweedelijnszorg vs 1.0 2012 and vs 2.0 2014.
3. Ohl CA, Dodds Ashley ES. Antimicrobial stewardship programs in community hospitals: the evidence base and case studies. Clin Infect Dis 2011;53(Suppl 1):23-28.
4. Phillips KW, Wittkowsky AK. Survey of pharmacist-managed inpatient anticoagulation services. Am J Health Syst Pharm 2007;64:2275-8.
5. Padron M, Miyares MA. Development of an Anticoagulation Stewardship Program at a Large Tertiary Care Academic Institution. J Pharm Pract 2013.
6. Winans AR, Rudd KM, Triller D. Assessing anticoagulation knowledge in patients new to warfarin therapy. Ann Pharmacother 2010;44(7-8):1152-7.
7. Moreland CJ, Kravitz RL, Paterniti DA, Li CS, Lin TC, White RH. Anticoagulation education: do patients understand potential medication-related emergencies? Jt Comm J Qual Patient Saf 2013;39(1):22-31.
8. Hylek EM, Held C, Alexander JH, Lopes RD, De Caterina R, Wojdyla DM, Huber K, Jansky P, Steg PG, Hanna M, Thomas L, Wallentin L, Granger CB. Major bleeding in patients with atrial fibrillation receiving apixaban or warfarin: The ARISTOTLE Trial (Apixaban for Reduction in Stroke and Other Thromboembolic Events in Atrial Fibrillation): Predictors, Characteristics, and Clinical Outcomes. Am Coll Cardiol. 2014;63(20):2141-7.
9. Gómez-Outes A, Terleira-Fernández AI, Calvo-Rojas G, Suárez-Gea ML, Vargas-Castrillón E. Dabigatran, Rivaroxaban, or Apixaban versus Warfarin in Patients with Nonvalvular Atrial Fibrillation: A Systematic Review and Meta-Analysis of Subgroups. Thrombosis 2013.
10. Deitelzweig SB, Pinsky B, Buysman E, Lacey M, Makenbaeva D, Wiederkehr D, Graham J. Bleeding as an outcome among patients with nonvalvular atrial fibrillation in a large managed care population. Clin Ther 2013;35(10).
11. DBC zorgproducten tariefapplicatie. [http://dbc-zorgproducten tarieven.nza.nl/nzaZpTarief].
12. Wille, N. et al. Development of the EQ-5D-Y: a child-friendly version of the EQ-5D. Quality of life research; 19(6): 885-886.
13. Ravens-Sieberer, U. et al. Feasibility, reliability, and validity of the EQ-5D-Y: results from a multinational study. Quality of life research 2010;19(6): 887-97.
14. EuroQol group: EuroQol-a new facility for the measurement of health-related quality of life. Health Policy 1990;16:199-208.
15. Horne R, Weinman J. Self-regulation and self-management in asthma: Exploring the role of illness perceptions and treatment beliefs in explaining non-adherence to preventer medication. *Psychology and Health 2002;*17(1): 17–32.
16. Ediger JP, Walker JR, Graff L, et al. Predictors of medication adherence in inflammatory bowel disease. *American Journal of Gastroenterology 2007;* 102(7): 1417–1426.
17. Horne R, Hankin M. The Medication Adherence Report Scale (MARS). In press

18. Thompson K, Kulkarni J, Sergejew AA. Reliability and validity of a new Medication Adherence Rating Scale (MARS) for the psychoses. Schizophr Res 2000;5:42(3):241-7.
19. Brokelman RBG, Haverkamp D. The validation of the visual analogue scale for patient satisfaction after total hip arthroplasty. Eur Orthop Traumatol. 2012;3:101-105.
20. WHO Collaborating Centre for Drug Statistics Methodology: Complete ATC index. 2009 [http://www.whocc.no/atcddd/].
21. Schulman S, Kearon C. Definition of major bleeding in clinical investigations of antihemostatic medicinal products in non-surgical patients. J Thromb Haemost 2005;3(4):692-4.
22. Schulman S, Angeras U. Definition of major bleeding in clinical investigations of antihemostatic medicinal products in surgical patients. J Thromb Haemost. 2010;8(1):202-4
23. Thygesen K, the Writing Group on behalf of the Joint ESC/ACCF/AHA/WHF Task Force for the Universal Definition of Myocardial Infarction. Third universal definition of myocardial infarction. European Heart Journal 2012;33:2551–2567.
24. Sacco RL, et al. An Updated Definition of Stroke for the 21st Century. *Stroke* 2013;44:2064-2089.
25. Ageno W, Squizzato A, Wells PS, Büller HR, Johnson G. The diagnosis of symptomatic recurrent pulmonary embolism and deep vein thrombosis: guidance from the SSC of the ISTH. J Thromb Haemost 2013;11(8):1597-602.
26. Division of Lung Diseases, National Heart, Lung, and Blood Institute. Value of the Ventilation/Perfusion Scan in Acute Pulmonary EmbolismResults of the Prospective Investigation of Pulmonary Embolism Diagnosis (PIOPED). *JAMA* 1990;263(20):2753-2759.
27. Rodeghiero F, Tosetto A, Abshire T, Arnold DM, Coller B, James P, Neunert C, Lillicrap D. ISTH/SSC bleeding assessment tool: a standardized questionnaire and a proposal for a new bleeding score for inherited bleeding disorders. J Thromb Haemost 2010;8(9):2063-5.
28. Shah NM, Hawwa AF. Adherence to antiepileptic medicines in children: A multiple methods assessment involving dried blood spot sampling. Epilepsia. 2013;54(6):1020-7.

ATTACHMENTS

1. Quality of life questionnaire: EQ5D and EQ5D-Y
2. Medication Adherence Rating Scale questionnaire: MARS5
3. Quality of care (patient satisfaction with antithrombotic therapy): VAS satisfaction scale
4. Questions on healthcare use
