# Supplementary figures and images for: Effect of antithrombotic stewardship on the efficacy and safety of antithrombotic therapy during and after hospitalization
Source: PLoS One. 2020 Jun 25;15(6):e0235048. doi: 10.1371/journal.pone.0235048 (PMC7316339; doi:10.1371/journal.pone.0235048)

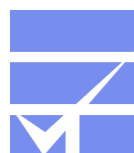

# CONSORT

TRANSPARENT REPORTING of TRIALS

## CONSORT 2010 Flow Diagram

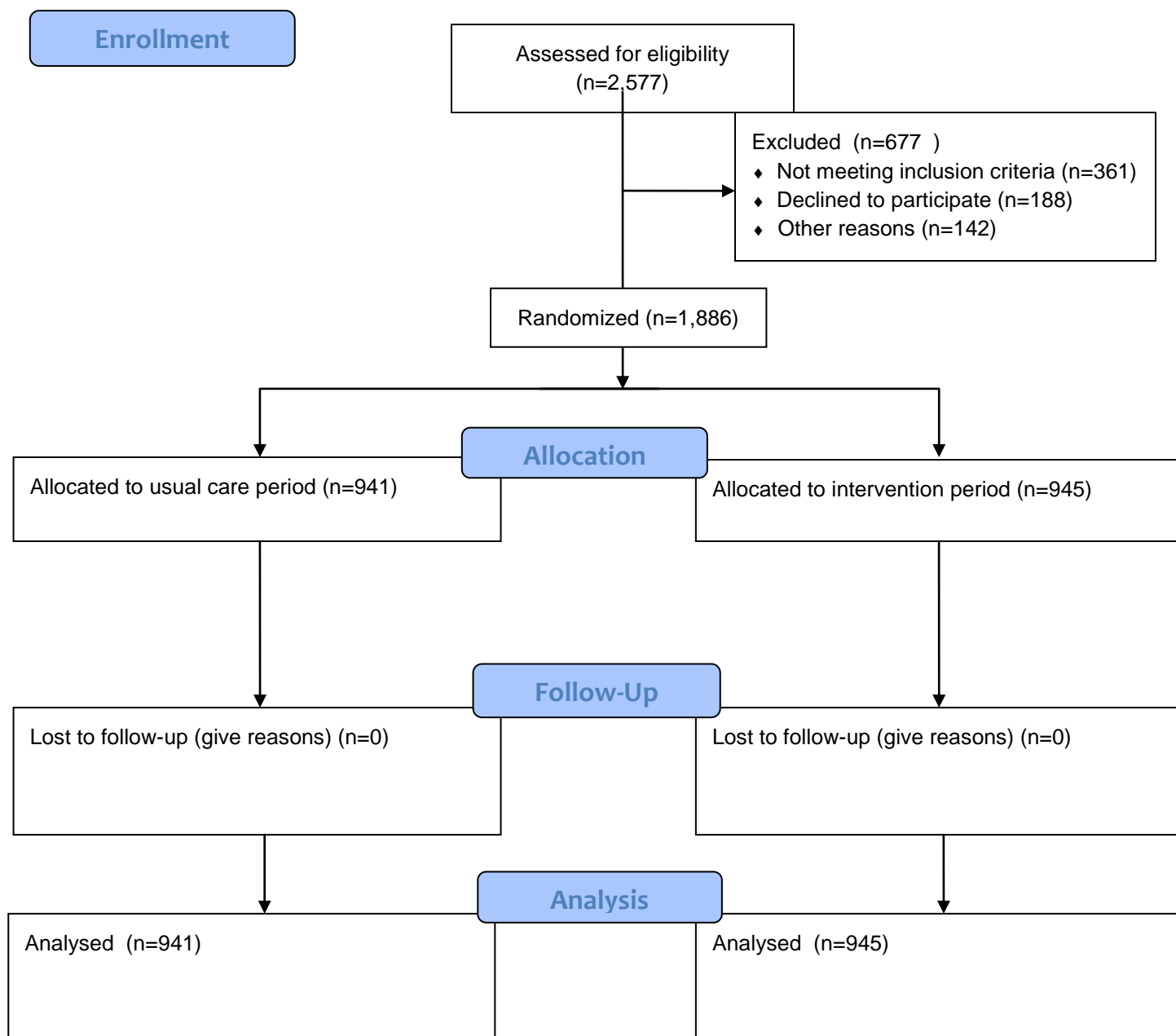

Supplement: S2 File — (PDF) [file pone.0235048.s008.pdf]
