# Supplementary material for: Effect of antithrombotic stewardship on the efficacy and safety of antithrombotic therapy during and after hospitalization
Source: PLoS One. 2020 Jun 25;15(6):e0235048. doi: 10.1371/journal.pone.0235048 (PMC7316339; doi:10.1371/journal.pone.0235048)
Supplement: S3 File — (DOCX) [file pone.0235048.s009.docx]

**Why was this study done?**

- Although the benefits of antithrombotic drugs are indisputable to reduce thrombotic events, they carry a high risk of compromising patient safety in terms of bleeding.
- Several studies have suggested that antithrombotic management initiatives improve patient outcomes, however, no impact on bleeding and thrombotic outcomes was observed.

**What did the researchers do and find?**

- We designed the S-team study (antithrombotic stewardship study; in Dutch: Stollingsteam), to study the effect of implementation of a hospital-based multidisciplinary antithrombotic team on the efficacy and safety of antithrombotic therapy during and after hospitalization.
- The team focused on education, medication reviews by pharmacists, implementing of local anticoagulant therapy guidelines based on national guidelines, patient counselling and medication reconciliation at admission and discharge.
- This study shows that implementation of a multidisciplinary antithrombotic team over time significantly reduces the composite end point consisting of one or more bleeding episodes or one or more thrombotic event from hospitalization until three months after hospitalization in patients using anticoagulant drugs.

**What do these findings mean?**

- The current findings show implementation of a multidisciplinary antithrombotic team was associated with a reduction in the proportion of patients with complications associated with the use of anticoagulant drugs.
