# Supplementary material for: Effect of antithrombotic stewardship on the efficacy and safety of antithrombotic therapy during and after hospitalization
Source: PLoS One. 2020 Jun 25;15(6):e0235048. doi: 10.1371/journal.pone.0235048 (PMC7316339; doi:10.1371/journal.pone.0235048)
Supplement: Attachment — Submitted filename: Responses to the reviewers comments.docx [file pone.0235048.s010.docx]

**Reviewer comments of PLOS ONE PONE-D-19-31280**

Effect of antithrombotic stewardship on the efficacy and safety of antithrombotic therapy during and after hospitalization

**Reviewer #1**

Interesting study with some technical merits.

However, there are major limits that should explained and discussed; additional statistics are required on adjustment analyses:

1. The author did not confirm their primary hypothesis of 9% composite before and 5.5% after intervention and there was no reduction at all based on this prevision.

Our study was powered to detect a total difference of 3.5% in the composite endpoint after our intervention and considering a 9% baseline prevalence rate. We observed a baseline rate of 9.49% (5.36 to 13.61) at start of the study based on the segmented regression model [Figure 2]. In addition, we observed a statistical significant change in the composite endpoint rate (see answer to Q4). We therefore, believe that we have confirmed our primary endpoint.

We have clarified the link between our observed results in relation to the assumptions in the power calculation in the results section of the manuscript. Page 13, lines 262-266.

“The segmented regression analysis showed that in the baseline period, i.e., before the introduction (baseline period) of the multidisciplinary team, the proportion of patients with a bleeding or thrombotic event at time=0 was 9.49% (5.36 to 13.61) that showed an increase of 0.75% per 2 months (0.23% to 1.28%) during the baseline period.”

2. No multivariable analysis was done (ie, no adjustment on variables differently distributed between the usual and the intervention group): this should be done, because there were strong differences in proportion of DOACs.

We have performed the multivariable analysis adjusted for characteristics differing between usual care and intervention period (bleeding in history and treatment with VKAs, DOACs or LMWHs). Table 2 on page 24 and Table S3.

We have added the multivariable analysis to the methods section (page 11, line 226) and the results sections (page 15, lines 299-302 and lines 313-315) of the manuscript.

3. Was there a predefined statistical plan analysis ? Was the use of segmental regression analysis pre-planned?

There was a predefined statistical plan and the use of segmental regression analysis was also pre-planned. We have added this to the methods section of the manuscript. Page 11, lines 205-206.

A detailed description of the statistical plan can be found in our previously published study protocol.

“Dreijer AR, Kruip MJHA, Diepstraten J, Polinder S, Brouwer R, Leebeek FWG, et al. Antithrombotic stewardship: a multidisciplinary team approach towards improving antithrombotic therapy outcomes during and after hospitalisation: a study protocol. BMJ Open. 2016;6:e011537.”

4. Finally, the conclusion should be lowered: the study suggests a potential improvement, but there is no demonstration.

An Interrupted Times Series study evaluates two aspects of changes caused by an intervention. First, it looks at an immediate change that occurs after the intervention, which in our study was not significant. The second part of the (segmented regression) analysis then looks if there is a gradual change over time. Our intervention showed that introduction of the multidisciplinary team indeed led to such a gradual change over time showing a significant reduction (-1.83% (-2.58% to -1.08%) per 2 months) in the proportion of patients with the composite end point consisting of one or more bleeding episodes or one or more thrombotic event from hospitalization until three months after hospitalization in patients using anticoagulant drugs. We based our conclusion on these findings and therefore did not adjust the conclusion.

5. The underlying disease that indicated anticoagulant therapy is not described; this is a major limitation since VTE and atrial fibrillation, for example, are not the same diseases at all and the thrombotic complications are different in terms of frequency and type of event.

Thank you for this observation. We have added the number of patients with atrial fibrillation, venous thromboembolism and cardiac calve surgery to the results section of the manuscript. Page 13, lines 255-257.

6. Table 2: a line with the total composite is needed in each period.

We have added the total composite of the type of anticoagulant therapy and hospital type to Table 2 on page 24.

7. In the flow chart, authors indicate randomization, which is wrong.

We have removed the term “randomization” in the flow chart in figure 1.

**Reviewer #2**

This is a truly relevant study. The evaluation is quite complete, including clinical and economic criteria. The implementation of a multidisciplinary team in charge of therapeutic education and medication review may prevent interactions, duplicate medications, and dosing errors. Therefore, we can assume it could reduce the thrombotic and bleeding events. I don’t see any major issue in the manuscript. You will find below a few minor suggestions.

1. Abstract, line 21: The primary endpoint could be described in the methods section.

We have added the primary outcome to the abstract section of the manuscript. Page 2, lines 18-20.

“Primary outcome was the proportion of patients with a composite end point consisting of one or more bleeding episodes or one or more thrombotic event from hospitalization until three months after hospitalization.”

2. Methods, line 171: Some other systemic embolisms could have been included. The decision to only include myocardial infraction and ischemic stroke as arterial thrombosis could be justified.

As described in our study protocol we also have included any objectively determined arterial or venous thrombus. We have clarified this and added it to the methods section of the manuscript. Page 10, lines 173-176.

3. Discussion, line 373-379: Considering NOACs have limited drug interactions, do not require routine coagulation monitoring with fixed doses, they are easier to use than VKA. Their use does not require a extensive experience.

We agree and have added the following sentence to the discussion section. Page 18, lines 393-395.

“However, as DOACs have limited drug interactions, they do not require coagulation monitoring. They are easier to use than VKAs and do not require extensive experience.”

4. Discussion, line 414: It might have been less occurrences of underreporting of the non-major bleeding events in the S-Team group. Indeed we can assume that after a therapeutic education, patients might have been more inclined to notify a non-major bleeding.

We have removed the sentence that we have no reason to assume that this underreporting occurred more often in either of the measurement periods. Page 20, lines 432-435.
