# Supplementary material for: Effect of antithrombotic stewardship on the efficacy and safety of antithrombotic therapy during and after hospitalization
Source: PLoS One. 2020 Jun 25;15(6):e0235048. doi: 10.1371/journal.pone.0235048 (PMC7316339; doi:10.1371/journal.pone.0235048)
Supplement: Attachment — Submitted filename: Response to Reviewers.docx [file pone.0235048.s011.docx]

**Reviewer comments of PLOS ONE PONE-D-19-31280R1**

Effect of antithrombotic stewardship on the efficacy and safety of antithrombotic therapy during and after hospitalization

**Reviewer #1**

All the questions have been addressed. I do not have further comments and the manuscript is suitable for publication.

**Reviewer #3**

The primary aim, as stated, of the prospective nonrandomized clinical trial was to evaluate the efficacy and safety of antithrombotic therapy during and after hospitalization. A group of patients received the intervention protocol while another group served as controls. Although a significant change in the proportion of patients with a thrombotic event was not apparent immediately, by 2 months a significant decrease was observed.

Minor revisions:

1. Abstract: Consider beginning the following sentence with the phrase, The primary aim was to estimate the proportion of patients ... “Primary outcome was the proportion
of patients ... with a composite end point consisting of one or more bleeding episodes or
one or more thrombotic event from hospitalization until three months after hospitalization.”

We have adjusted the sentence in the Abstract section of the manuscript. Page 2, lines 19-21.

“The primary aim was to estimate the proportion of patients with a composite end point consisting of one or more bleeding episodes or one or more thrombotic event from hospitalization until three months after hospitalization”.

2. Abstract: Provide more clarity in the following statement. Specifically identify the primary endpoint. “Introduction of the multidisciplinary team had no immediate impact +1.63% (-3.60% to +6.85%) on the primary endpoint, but over time the primary endpoint event rate decreased significantly with -1.83% (-2.58% to -1.08%) per 2 month period.”

We have clarified the results in the Abstract section. Page 2 and 3, lines 28-32.

“The S-team study showed that implementation of a multidisciplinary antithrombotic team over time significantly reduced the composite end point consisting of one or more bleeding episodes or one or more thrombotic event from hospitalization until three months after hospitalization in patients using anticoagulant drugs (-1.83% (-2.58% to -1.08%) per 2 month period)”

3. Line 190: Indicate the statistical testing method which attains 80% power.

Because a sample size calculation was not possible for an interrupted time series analysis, we have used the Chi Square test on the advice of the statistician. We have indicated the statistical testing method in the sample size section of the manuscript. Page 10, lines 195-197.

“A Chi Square test with a type 1 error of 0.05, power 80% resulted in a sample size of 1,834 patients.”

4. Line 232: Clarify the type of subgroup analysis performed.

We have clarified the type of subgroup analyses. Page 12, lines 235-239.

“Subgroup analyses were performed on the proportion of patients with a composite end point consisting of one or more bleeding or one or more thrombotic event from hospitalization until three months after hospitalization stratified for each type of antithrombotic treatment (VKA, DOAC and LMWH) and for hospital type (Reinier de Graaf Gasthuis and the Erasmus University Medical Center).”

5. Line 282: Clarify that the numbers in parentheses represent the confidence interval.

We have clarified the numbers in parentheses representing the confidence intervals. Page 14, lines 284-288.

“Implementation of the multidisciplinary antithrombotic team showed no significant effect on the proportion of patients with a major bleeding event between the usual care period and intervention period (odds ratio [OR] 0.77; 95% confidence interval [95% CI] 0.55-1.10). The same applies to the proportion of patients with non-major bleeding events (OR 1.40; 95% CI 0.90-2.10) before and after implementation of the multidisciplinary antithrombotic team”

6. Table 3: Provide a measure of dispersion corresponding to each mean costs.

We have added the interquartile ranges (IQR) to the mean costs in Table 3 on page 25.

7. Provide a summary of adverse events to address the safety aim.

Because bleeding and thrombotic events are the most important adverse events of antithrombotic therapy we have chosen to register only that type of adverse event. Characterization of all bleeding and thrombotic events before and after implementation of the multidisciplinary antithrombotic team are listed in Table S2.

8. Identify the sources of funding for the study.

The sources of funding for this study are described on page 22, lines 488-493.

“Stichting Phoenix Schiedam, the pharmaceutical companies (Daiichi Sankyo, Boehringer Ingelheim, Bayer and Pfizer) and the Scientific Committee Reinier de Graaf Gasthuis provided financial support for this study in the form of unrestricted grants. The funders had no role in study design, data collection and analysis, decision to publish, or preparation of the manuscript and this does not alter our adherence to PLOS ONE policies on sharing data and materials.”

9. The primary aim of the study does not appear to be to determine efficacy and safety, but to compare the proportion of patients in the two groups who develop thrombotic events. Please clarify.

We have adjusted the primary aim in the background section of the abstract. Page 2, lines 12-15.

“The primary aim of this study was to compare the proportion of patients with a composite end point consisting of one or more bleeding episodes or one or more thrombotic event from hospitalization until three months after hospitalization.”
